# Resistin concentrations in perivascular adipose tissue as a highly sensitive marker of smoking status in patients with advanced coronary artery disease requiring coronary artery bypass grafting

**DOI:** 10.3389/fpubh.2024.1484195

**Published:** 2024-11-20

**Authors:** Maciej Rachwalik, Przemysław Sareło, Marta Obremska, Małgorzata Matusiewicz, Kaung Sithu Sett, Michał Czapla, Marek Jasiński, Magdalena Hurkacz

**Affiliations:** ^1^Department of Cardiac Surgery and Heart Transplantation, Institute of Heart Diseases, Wrocław Medical University, Wrocław, Poland; ^2^Department of Biomedical Engineering, Faculty of Fundamental Problems of Technology, Wrocław University of Science and Technology, Wrocław, Poland; ^3^Pre-clinical Research Center, Wrocław Medical University, Wrocław, Poland; ^4^Department of Cardiovascular Imaging, Institute of Heart Diseases, Wrocław Medical University, Wrocław, Poland; ^5^Division of Medical Biochemistry, Department of Biochemistry and Immunochemistry, Wroclaw Medical University, Wrocław, Poland; ^6^Student, Faculty of Medicine, Wrocław Medical University, Wrocław, Poland; ^7^Division of Scientific Research and Innovation in Emergency Medical Service, Department of Emergency Medical Service, Faculty of Nursing and Midwifery, Wroclaw Medical University, Wrocław, Poland; ^8^Group of Research in Care (GRUPAC), Faculty of Health Sciences, University of La Rioja, Logroño, Spain; ^9^Department of Clinical Pharmacology, Faculty of Pharmacy, Wrocław Medical University, Wrocław, Poland

**Keywords:** resistin, smoking, adipose tissue, coronary artery disease, coronary artery bypass grafting

## Abstract

**Background:**

Smoking is a significant risk factor for numerous diseases, including coronary artery disease (CAD). Chronic inflammation from smoking affects endothelial function and may alter adipokine secretion, particularly resistin, in perivascular adipose tissue (PVAT). This study investigated the association between resistin concentrations in PVAT and smoking status in CAD patients undergoing coronary artery bypass grafting (CABG).

**Methods:**

The study included 110 patients with advanced CAD scheduled for CABG. Patients were categorized into never-smokers and ever-smokers, with the latter further divided into current and past smokers. Resistin concentrations in PVAT and plasma, along with plasma interleukin-6 (IL-6) and high-sensitivity C-reactive protein (hs-CRP) concentrations, were measured using ELISA.

**Result:**

Significant differences in PVAT resistin concentrations were observed between never-smokers and ever-smokers (*p* < 0.0001), as well as between never-smokers and both current (*p* < 0.0001) and past smokers (*p* < 0.0001). PVAT resistin concentrations correlated positively with the number of pack-years (*p* < 0.0001) and plasma resistin (*p* < 0.0001) and IL-6 concentrations (*p* < 0.0001). Plasma resistin, IL-6, and hs-CRP concentrations were higher in ever-smokers compared with never-smokers. Multiple regression analysis indicated that smoking is significantly correlated with higher PVAT resistin concentrations, with increased pack-years (*p* = 0.0002), higher plasma resistin concentrations (*p* < 0.0001), and IL-6 concentrations (*p* < 0.0001), all contributing to elevated PVAT resistin.

**Conclusion:**

Smoking status in advanced CAD patients requiring CABG is positively associated with PVAT resistin concentrations, with a clear demonstration of dose-dependency.

## 1 Introduction

It is well known that smoking is associated with significant health risks. Nevertheless, tobacco use remains high in developing countries. It was estimated that smoking causes the premature death of nearly 8 million people worldwide each year, including about 15% of nonsmokers who die from passive smoking (1).

Chronic cigarette smoking has also been linked to the development of numerous cardiovascular and respiratory diseases, as well as various types of cancer (2). Numerous reports have described a strong association between smoking and chronic systemic inflammation, defined as an imbalance of cytokines and other proinflammatory mediators (3, 4). Chronic inflammation and abnormal levels of proinflammatory factors alter endothelial cell function, and endothelial cells play a critical role in vascular wall homeostasis (5, 6).

Recently, specific interactions between vessel wall cells and perivascular adipose tissue (PVAT) have been reported, which occur due to their proximity to the arterial wall (7, 8). PVAT forms the outer layer of the vessel wall and is composed of terminally undifferentiated adipocytes. It is responsible for multidirectional actions, including the regulation of vascular tone, intravascular thermoregulation, vascular smooth muscle cell proliferation, and, importantly, it has a significant endocrine-paracrine function.

The secretory activity of PVAT can be altered by several pathophysiological and clinical conditions (8). Substantial evidence indicates that smoking is one of the main potential drivers of pathological processes in adipose tissue. Therefore, it was reported that PVAT disorders caused by smoking may accelerate the progression of atherosclerosis through proinflammatory endothelial activation through adipose-derived factors (9–11). One of the adipokines which PVAT secretes is resistin, which can be attributed to local vasculature inflammation. Resistin in plasma is mainly secreted by bone marrow, monocytes, and macrophages (12–14). Specific proinflammatory properties have been linked with the development of insulin resistance, type 2 diabetes, atherosclerosis, and cardiovascular disease (15–18). Resistin in plasma is a subclinical marker of atherosclerosis, and its elevated concentrations is associated with increased mortality (19, 20). Furthermore, an increased concentrations of resistin in plasma is observed in active smokers (20–22). However, the relationship between smoking and resistin concentrations in PVAT has not been fully elucidated.

The aim of this study was to evaluate the associations between resistin concentrations in PVAT and proinflammatory status based on serum resistin, IL-6, and high-sensitivity C-reactive protein (hs-CRP) concentrations in patients with confirmed advanced CAD requiring coronary artery bypass grafting (CABG), depending on smoking status.

## 2 Methods

### 2.1 Characteristics of the study cohort

Patients were recruited between February 2017 and December 2022. During this period, a total of 2,548 myocardial revascularization procedures were performed at the Department of Cardiac Surgery and Heart Transplantation of the Institute of Heart Diseases at Wroclaw Medical University Hospital in Wrocław, Poland. A total of 212 consecutive patients who underwent CABG during this period, performed by the author of this publication, were considered for inclusion in the study. Of these, 128 patients met the inclusion criteria and provided informed consent to participate. Due to technical difficulties with the procedures or patients withdrawing their consent to participate in the study, the final sample included 110 patients.

Before the qualification to CABG, all patients underwent a coronary angiography and an echocardiography. However, before the surgical procedure, laboratory tests, diabetic treatment and pharmacotherapy were reviewed. Inclusion criteria were as follows: planned myocardial revascularization with extracorporeal circulation, sinus rhythm on electrocardiography, left ventricular ejection fraction higher than 50% on echocardiography, age less than 80 years, and patient consent to participate in the study. Exclusion criteria were severe or moderate valvular heart disease or other heart diseases requiring cardiac surgery (such as aortic or left ventricular aneurysm), history of atrial fibrillation, end-stage renal disease, rheumatic disease, and cancer.

Patients received standard pharmacotherapy including *β*-blockers, angiotensin-converting enzyme inhibitors, statins (rosuvastatin in all cases), nitrates, subcutaneous low-molecular-weight heparin, and aspirin. All patients undergoing CABG were not taking oral inhibitors of sodium-dependent glucose transporter 2. Patients with diabetes were treated with metformin, which was discontinued 2 days before surgery. Five patients with diabetes were receiving subcutaneous insulin. Drugs were administered according to pharmacotherapy guidelines and adjusted for patient weight and clinical condition.

### 2.2 Blood sample collection and biochemical analyses

Venous blood samples were collected into serum clot activator vacuum tubes (BD, Franklin Lakes, New Jersey, USA) before CABG to determine serum concentrations of selected biomarkers, including resistin and IL-6. Samples were centrifuged in a laboratory centrifuge at 5000 rpm for 10 min. The obtained sera were frozen at −80°C.

Other biochemical parameters, such as hs-CRP, total cholesterol (TC), high-density lipoprotein cholesterol (HDL-C), low-density lipoprotein cholesterol (LDL-C), triglycerides (TG), glucose, and creatinine [to calculate estimated glomerular filtration rate (eGFR)] were determined before surgery in the hospital laboratory using standard diagnostic methods with the AU480 (Beckman Coulter, Brea, California, USA) and Cobas Integra 400 plus (Hoffmann-La Roche, Basel, Switzerland) biochemical analyzers using photometry, turbidimetry, and potentiometry.

### 2.3 Coronary artery bypass grafting

Standard sternotomy was used for CABG procedures. Extracorporeal circulation was performed using an aortic cannula inserted into the ascending aorta and a venous return cannula inserted into the right atrial appendage. The average duration of aortic cross-clamping was 40–45 min. The duration of aortic cross-clamping during CABG and postoperative troponin concentrations were noted.

### 2.4 Perivascular adipose tissue sample collection and resistin concentrations assessment

After cardiac arrest, PVAT around the left main coronary artery was excised using surgical scissors and a blade. A small portion of the sample (3 × 3 × 3 mm) was immediately frozen at −80°C and stored for further analysis. The mean weight of PVAT samples collected during the procedure was 0.0303 ± 0.0166 g.

A FastPrep-24 homogenizer (MP Biomedicals, Santa Ana, California, USA) was used to homogenize the PVAT samples, and then phosphate-buffer saline (Sigma-Aldrich, St. Louis, Missouri, USA) was used. Centrifugation was performed twice for 10 min at 14000 × *g* and a temperature of 4°C. The second centrifugation was performed just before the analysis. PVAT and plasma resistin concentrations were measured using the Human Resistin Quantikine ELISA kit (R&D Systems, Minneapolis, Minnesota, USA) according to the manufacturer’s instructions and were expressed as ng/g of tissue or ng/mL of plasma. IL-6 concentrations were measured using the Human IL-6 Quantikine ELISA kit (R&D Systems).

### 2.5 Patient subgroups

**Figure 1:**
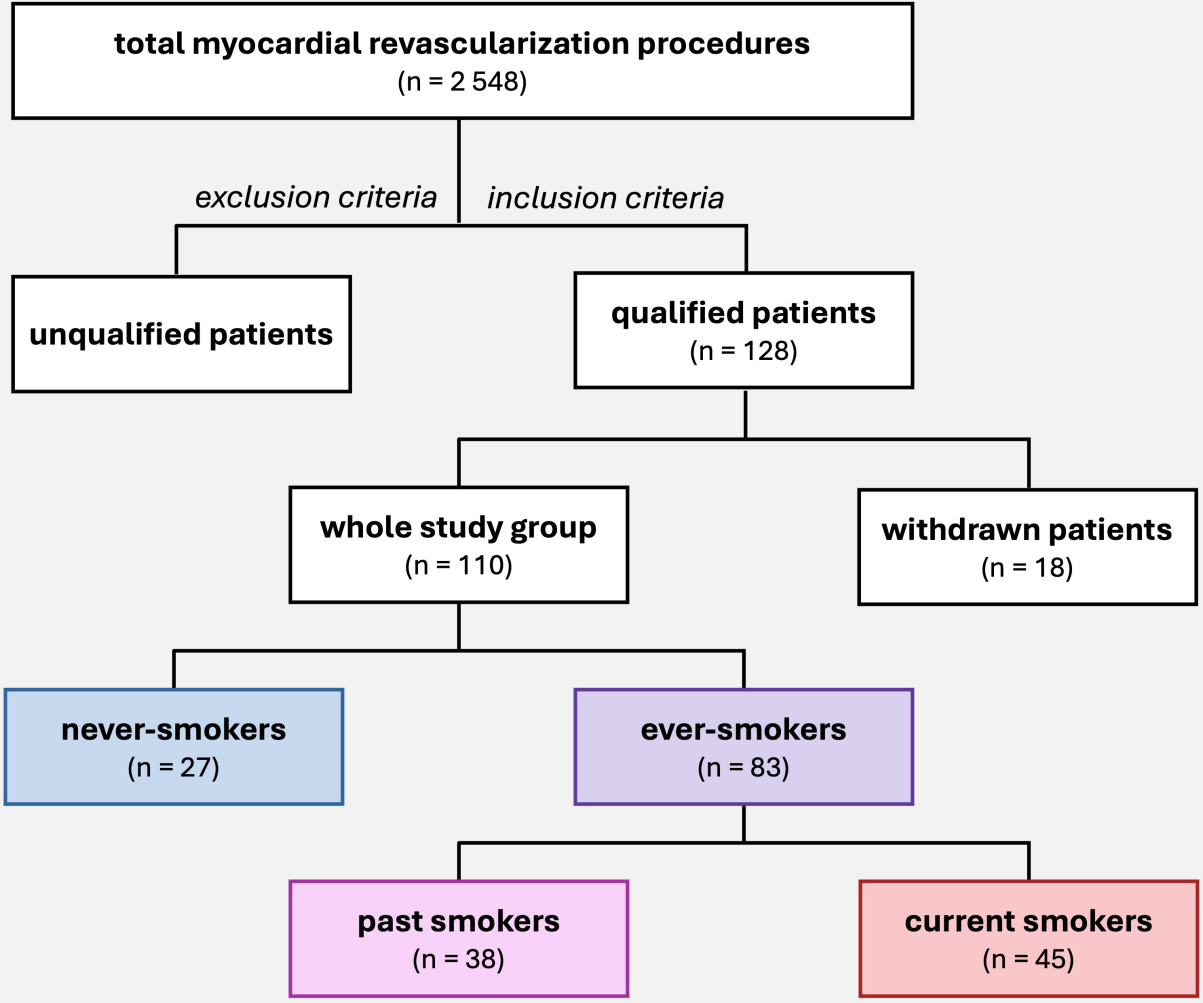
Patient selection for the study and subgroup division according to smoking status. A total of 2,548 myocardial revascularization procedures were performed at the Department of Cardiac Surgery between 2017 and 2022. The study initially included 128 patients who were operated by author of study and met the inclusion criteria. During the study, 18 patients were withdrawn, and the final group consisted of 110 patients, who were then divided into subgroups based on smoking habits.

A total of 110 patients were included in the final analysis at mean age 66.8 ± 7.9, male 75.5%. Based on an original survey of current and past smoking habits, the population was divided into 2 groups (Figure 1): ever-smokers and never-smokers. Never-smokers were patients who had never smoked cigarettes. The group of ever-smokers was further divided into current smokers and past smokers. Current smokers were patients who continued to smoke within the year prior to surgery. Past smokers were patients who had quit smoking at least 1 year before surgery. Smoking was quantified and expressed in pack-years. The study population consisted of 27 never-smokers and 83 ever-smokers (including 45 current smokers and 38 past smokers) (Figure 1).

### 2.6 Statistical analysis

Statistical analysis was conducted using Statistica 13.3 (TIBCO Software, Palo Alto, California, USA) and GraphPad Prism 10 (Dotmatics, Boston, Massachusetts, USA).

The Shapiro–Wilk test was used to determine the normality of data distribution. The Levene’s test was used to assess whether data met the criterion of equality of variance. A nonparametric approach was used to compare never-smokers and ever-smokers, because the assumptions of normality of distribution and homogeneity of most variances were not met. The Mann–Whitney U test was used for the comparison. The nonparametric Kruskal-Wallis and Dunn’s multiple comparison tests were used to compare the study group (never-smokers) with the study subgroups (past smokers and current smokers). To examine the significance of the association (contingency) between the two types of classification (i.e., diabetes vs. no diabetes) in the never-smoker and ever-smoker groups as well as in the past and current smoker subgroups, the Fisher exact test was used. Additionally, Spearman’s rank correlation test was applied to assess the strength of the relationship between variables. Multivariate analysis was performed to determine relationships between different variables. A *p*-value of less than 0.05 was considered significant.

## 3 Results

### 3.1 Differences in clinical and demographic characteristics between never-smokers and ever-smokers

The demographic and clinical characteristics of never-smokers and ever-smokers are presented in Table 1.

**Table 1:** Demographic and clinical characteristics of never-smokers and ever-smokers.

Variable	Never-smokers (*n* = 27)				Ever-smokers (*n* = 83)				*p-*value*
	Mean	SD	Median	IQR	Mean	SD	Median	IQR	
Age (years)	66.9	6.9	68.0	8.5	66.2	8.2	66.0	9.0	0.3569
Body weight (kg)	82.1	13.2	82.5	14.8	80.9	17.5	82.0	20.3	0.9680
Height (m)	1.7	0.1	1.7	0.1	1.7	0.1	1.7	0.2	0.6297
BMI (kg/m^2^)	28.4	4.2	27.5	3.6	28.0	4.3	28.3	4.7	0.9424
PVAT resistin (ng·g^−1^)	17.12	14.05	14.80	5.98	51.9	37.0	45.0	34.3	<0.0001*
Plasma resistin (ng·mL^−1^)	2.56	1.25	2.22	1.48	4.35	2.36	4.00	3.20	0.0002*
IL-6 (pg·mL^−1^)	3.64	4.45	2.50	1.65	7.61	9.23	3.04	6.09	0.0166*
hs-CRP (mg·L^−1^)	18.26	54.20	2.60	3.96	18.71	38.66	9.14	14.90	0.0005*
TC (mg·dL^−1^)	152.2	25.6	151.0	23.0	160.9	47.3	144.0	70.0	0.8016
HDL-C (mg·dL^−1^)	41.1	10.3	39.0	13.5	40.1	12.5	38.5	15.5	0.6691
LDL-C (mg·dL^−1^)	84.4	24.3	81.0	30.0	86.5	42.4	75.0	45.0	0.5947
TG (mg·dL^−1^)	138.8	43.4	139.5	55.8	145.5	93.2	124.5	69.0	0.6499
eGFR (mL·min^−1^·m^−2^)	70.42	16.44	69.00	24.50	75.8	22.4	77.0	24.5	0.1447
Glucose (mg·dL^−1^)	120.5	39.4	111.0	21.0	125.0	64.0	106.0	37.0	0.8103
Hb (g·dL^−1^)	14.0	1.1	14.0	1.3	13.6	1.7	13.6	1.9	0.3365

^*^p-values were calculated using the Mann–Whitney U test.

BMI, body mass index; Hb, hemoglobin; HDL-C, high-density lipoprotein cholesterol; hs-CRP, high-sensitivity C-reactive protein; eGFR, estimated glomerular filtration rate; IL-6, interleukin 6; LDL-C, low-density lipoprotein cholesterol; PVAT, perivascular adipose tissue; TC, total cholesterol; TG, triglycerides.

**Figure 2:**
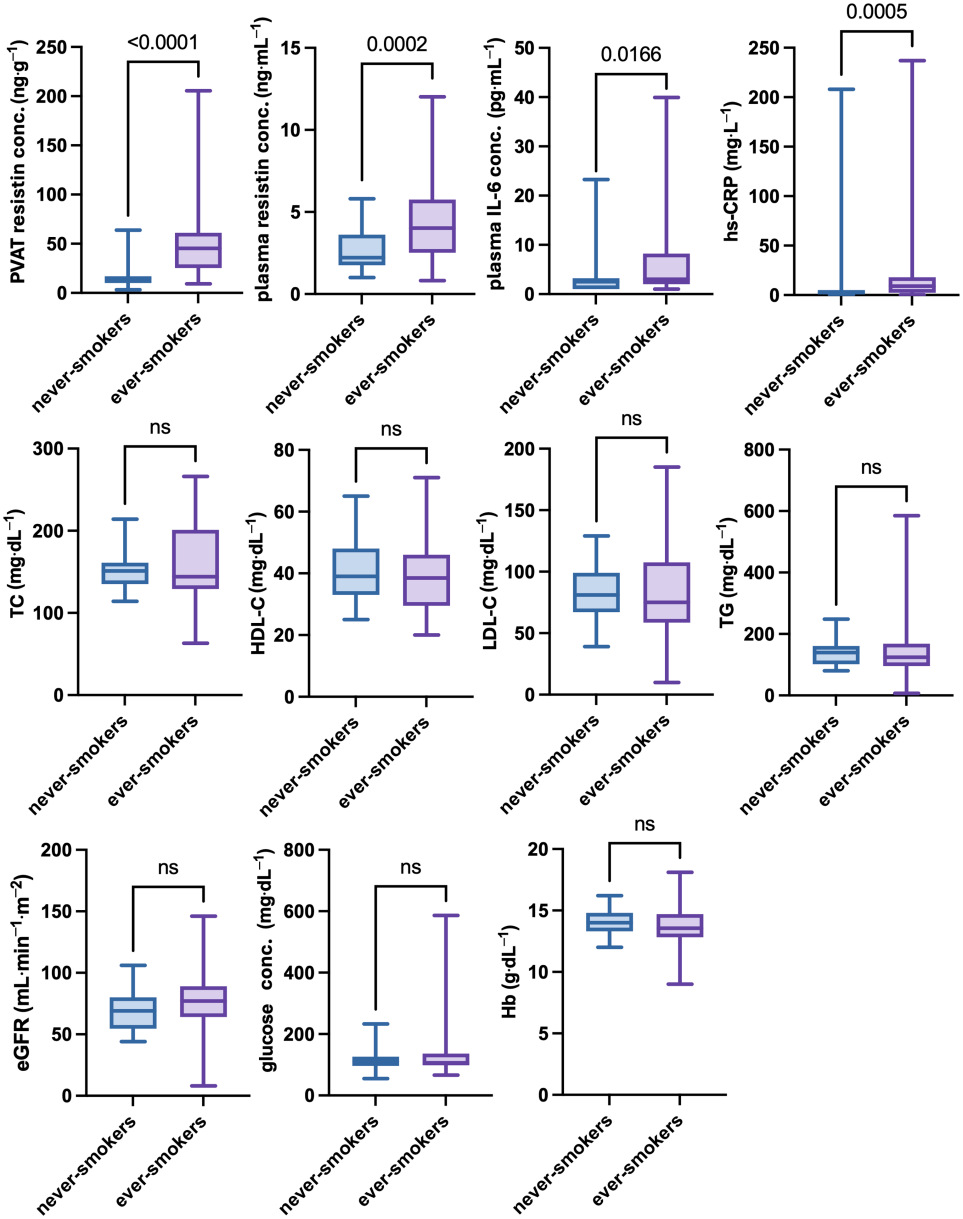
Differences in selected clinical parameters between never-smokers and ever-smokers. The group of ever smokes showed significantly higher concentrations of inflammatory parameters, such as resistin concentrations in PVAT and plasma, as well as IL-6 and CRP concentrations in plasma No statistically significant differences between never-smokers and ever-smokers in eGFR and plasma lipids, glucose, or hemoglobin concentration were found. A *p*-value of less than 0.05 was considered significant (nonparametric Mann–Whitney U test). eGFR, estimated glomerular filtration rate; Hb, hemoglobin; HDL-C, high-density lipoprotein cholesterol; hs-CRP, high-sensitivity C-reactive protein; IL-6, interleukin 6; LDL-C, low-density lipoprotein cholesterol; ns, nonsignificant; PVAT, perivascular adipose tissue; TC, total cholesterol; TG, triglycerides.

There were no significant differences in age, body weight, height, and body mass index (BMI) between never-smokers and ever-smokers. However, mean resistin concentrations in PVAT differed significantly between groups. Similarly, the groups differed in mean plasma resistin, IL-6, and hs-CRP concentrations. On the other hand, there were no significant differences in lipid profile, including TC, HDL-C, LDL-C, and TG concentrations, or in eGFR, glucose, and hemoglobin concentrations. The results are presented in Table 1 and Figure 2.

**Figure 3:**
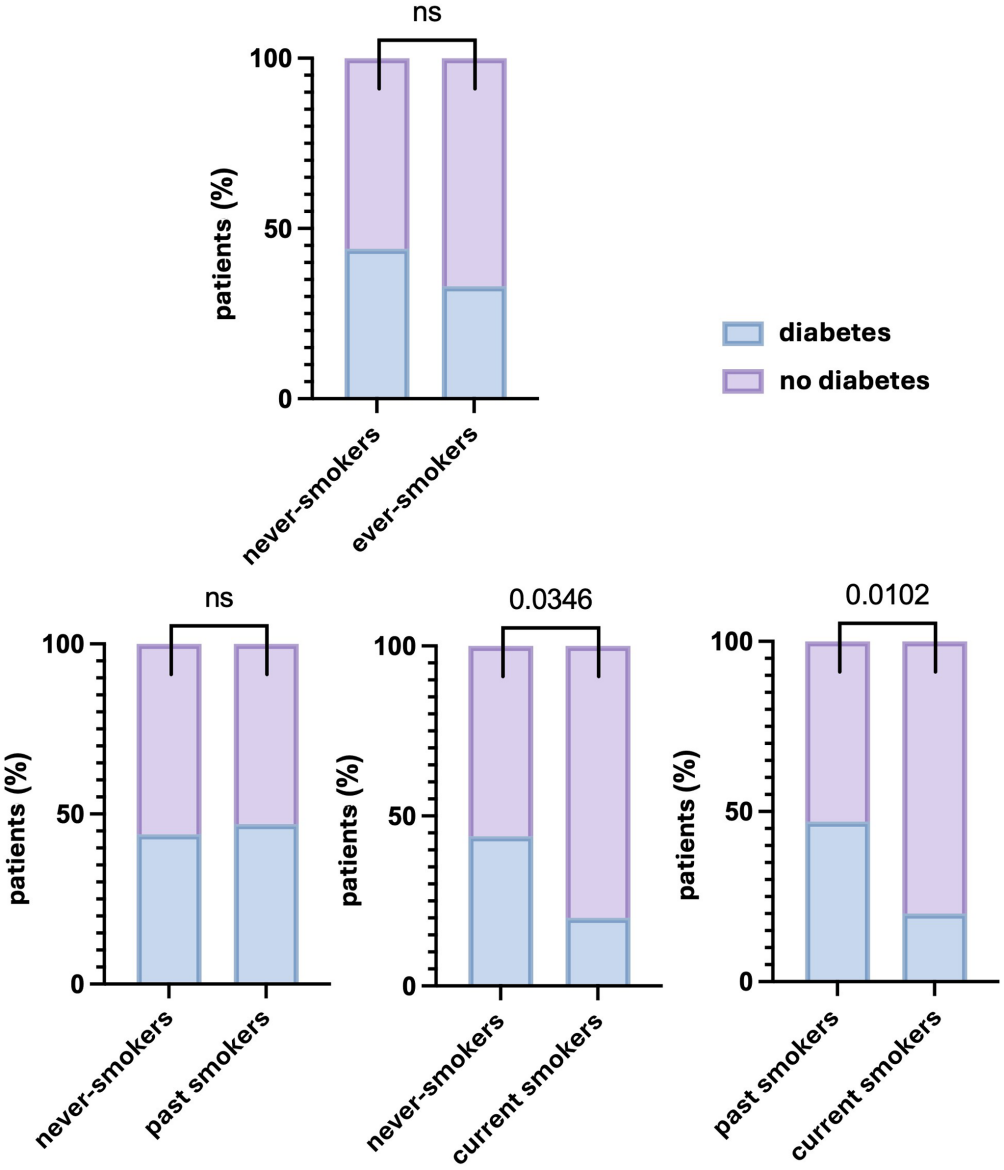
Differences in the percentage of patients with and without diabetes depending on the smoking status. Significant differences in the percentage of patients with diabetes were found between never-smokers and current smokers, as well as between past smokers and current smokers, but not between never-smokers and past smokers or ever-smokers. A *p* values of less than 0.05 was considered significant (Fisher’s exact test). ns, nonsignificant.

There were significant differences in the percentage of patients with and without diabetes between never-smokers and current smokers and between past smokers and current smokers. However, no significant differences were observed between never-smokers and past smokers (*p* > 0.9999). Data are presented in Figure 3. The demographic and clinical characteristics of past smokers and current smokers are presented in Table 2.

**Table 2:** Demographic and clinical characteristics of past smokers and current smokers.

Variable	Past smokers (*n* = 38)				Current smokers (*n* = 45)				*p*-value*
	Mean	SD	Median	IQR	Mean	SD	Median	IQR	
Age (years)	66.8	8.5	66.0	8.0	66.7	8.0	65.5	8.3	0.5669
Body weight (kg)	79.5	19.0	79.0	20.7	82.0	16.3	83.5	15.5	0.8351
Height (m)	1.7	0.1	1.7	0.2	1.7	0.1	1.7	0.1	0.7396
BMI (kg/m^2^)	28.3	4.0	28.7	4.6	27.7	4.6	28.3	5.2	0.8536
PVAT resistin (ng·g^−1^)	49.44	35.8	43.5	30.0	53.94	38.23	52.71	40.93	<0.0001
Plasma resistin (ng·mL^−1^)	4.56	2.06	4.51	1.89	4.19	2.59	3.78	3.80	0.0005
Plasma IL-6 (pg·mL^−1^)	5.35	6.47	2.99	2.54	9.47	10.72	5.00	11.18	0.0275
hs-CRP (mg·L^−1^)	16.90	34.47	9.57	14.70	20.24	42.21	9.01	13.00	0.0028
TC (mg·dL^−1^)	174.0	41.1	165.0	63.0	144.6	50.5	131.0	37.5	0.0149
HDL (mg·dL^−1^)	42.9	12.7	41.0	9.0	36.5	11.5	34.0	18.0	0.2103
LDL (mg·dL^−1^)	96.1	41.6	81.0	56.5	72.9	40.9	66.0	21.0	0.0285
TG (mg·dL^−1^)	160.0	112.2	125.0	59.5	125.8	56.0	122.0	81.0	0.8346
eGFR (mL·min^−1^·m^−2^)	71.7	19.3	72.5	18.3	79.2	24.4	80.0	22.5	0.0562
glucose (mg·dL^−1^)	126.2	44.2	105.0	52.8	124.0	77.2	107.0	21.5	0.9551

^*^p-values were calculated using the Kruskal-Wallis test to compare past smokers and current smokers.

**Figure 4:**
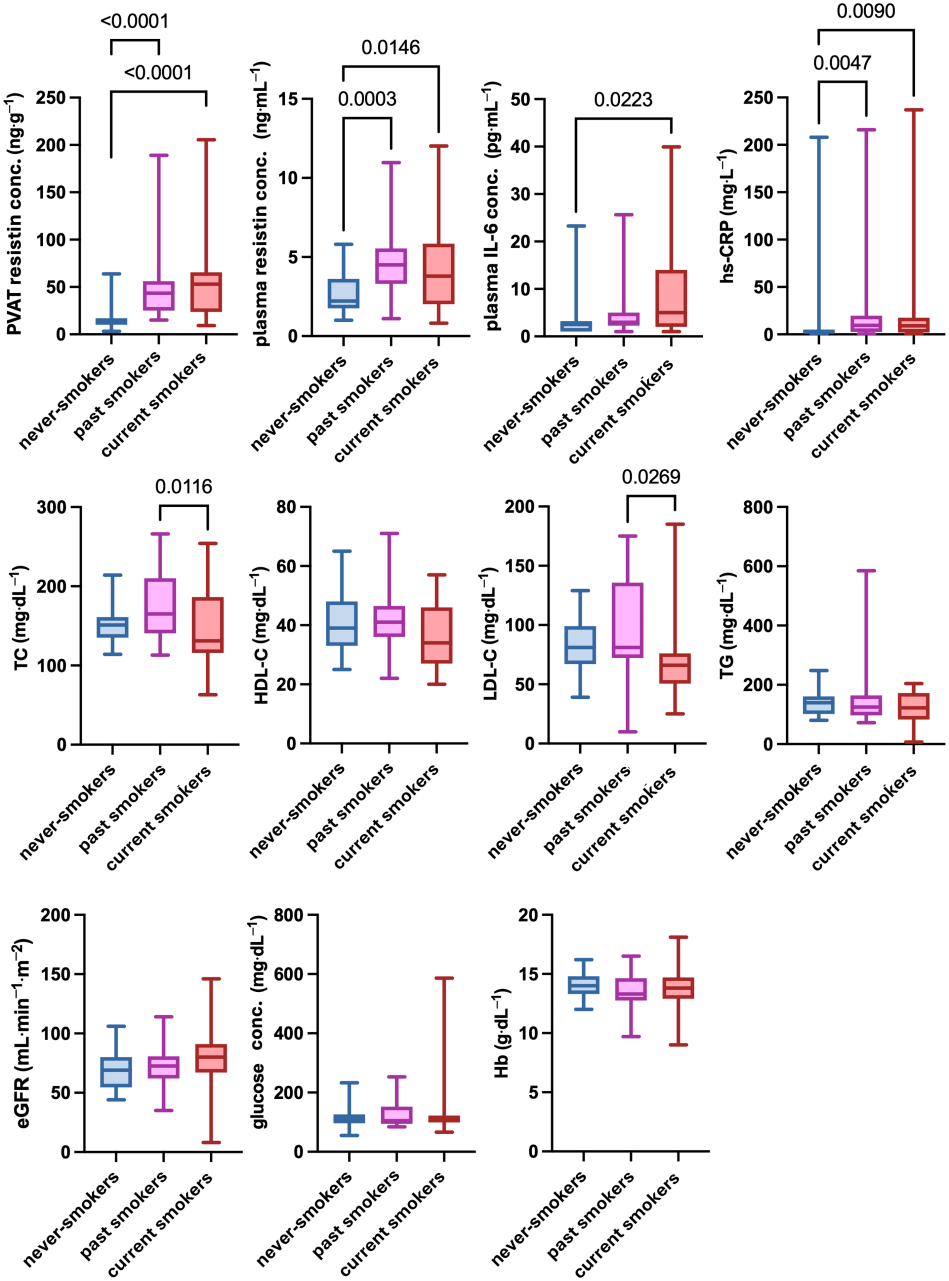
Differences in selected clinical parameters between never-smokers, past smokers, and current smokers. Significantly higher concentrations of resistin in PVAT and plasma, as well as plasma concentration of IL-6 and CRP, were found in both past smokers and current smokers compared to never-smokers. The plasma concentrations of TG and LDL-C were found to be significantly lower in current smokers compared to past smokers. A *p*-value of less than 0.05 was considered significant (nonparametric Kruskal-Wallis test and Dunn’s multiple comparisons test). eGFR, estimated glomerular filtration rate; Hb, hemoglobin; HDL-C, high-density lipoprotein cholesterol; hs-CRP, high-sensitivity C-reactive protein; IL-6, interleukin 6; LDL-C, low-density lipoprotein cholesterol; ns, nonsignificant; PVAT, perivascular adipose tissue; TC, total cholesterol; TG, triglycerides.

PVAT resistin concentrations differed between never-smokers and the groups of past and current smokers (Kruskal-Wallis test, *p* < 0.0001) (Table 2). The Dunn’s multiple comparisons test confirmed significant differences for never-smokers vs. past smokers (*p* < 0.0001) and never-smokers vs. current smokers (*p* < 0.0001). There were no differences between past and current smokers (*p* > 0.9999). Data are presented in Figure 4A.

Significant differences in plasma IL-6 and hs-CRP concentrations were found between never-smokers and past and current smokers (Kruskal-Wallis test, *p* = 0.0275 and *p* = 0.0028, respectively) (Table 2). Based on multiple comparisons, the differences in IL-6 concentrations were significant only between never-smokers and current smokers (*p* = 0.0223) (Figure 4A). On the other hand, hs-CRP concentrations differed significantly both between never-smokers and past smokers (*p* = 0.0047) and between never-smokers and current smokers (*p* = 0.0090) (Figure 4A).

In terms of the lipid profile, significant differences were found for TC and LDL-C concentrations between past smokers and current smokers (Kruskal-Wallis test, *p* = 0.0149 and *p* = 0.0285, respectively) (Table 2). In multiple comparisons, the *p*-value was 0.0116 for TC and 0.0269 for LDL-C. The remaining lipid profile parameters did not show significant differences between the subgroups (Figure 4B).

No significant differences were shown for eGFR, glucose, and hemoglobin concentrations between never-smokers and past and current smokers (*p* = 0.0562, *p* = 0.9551, and *p* = 0.5331, respectively). Respectively, in the case of eGFR, a trend toward higher values was observed in the group of smokers. The results are presented in Figure 4C.

### 3.2 Spearman’s rank correlation analysis of selected parameters in ever-smokers

**Figure 5:**
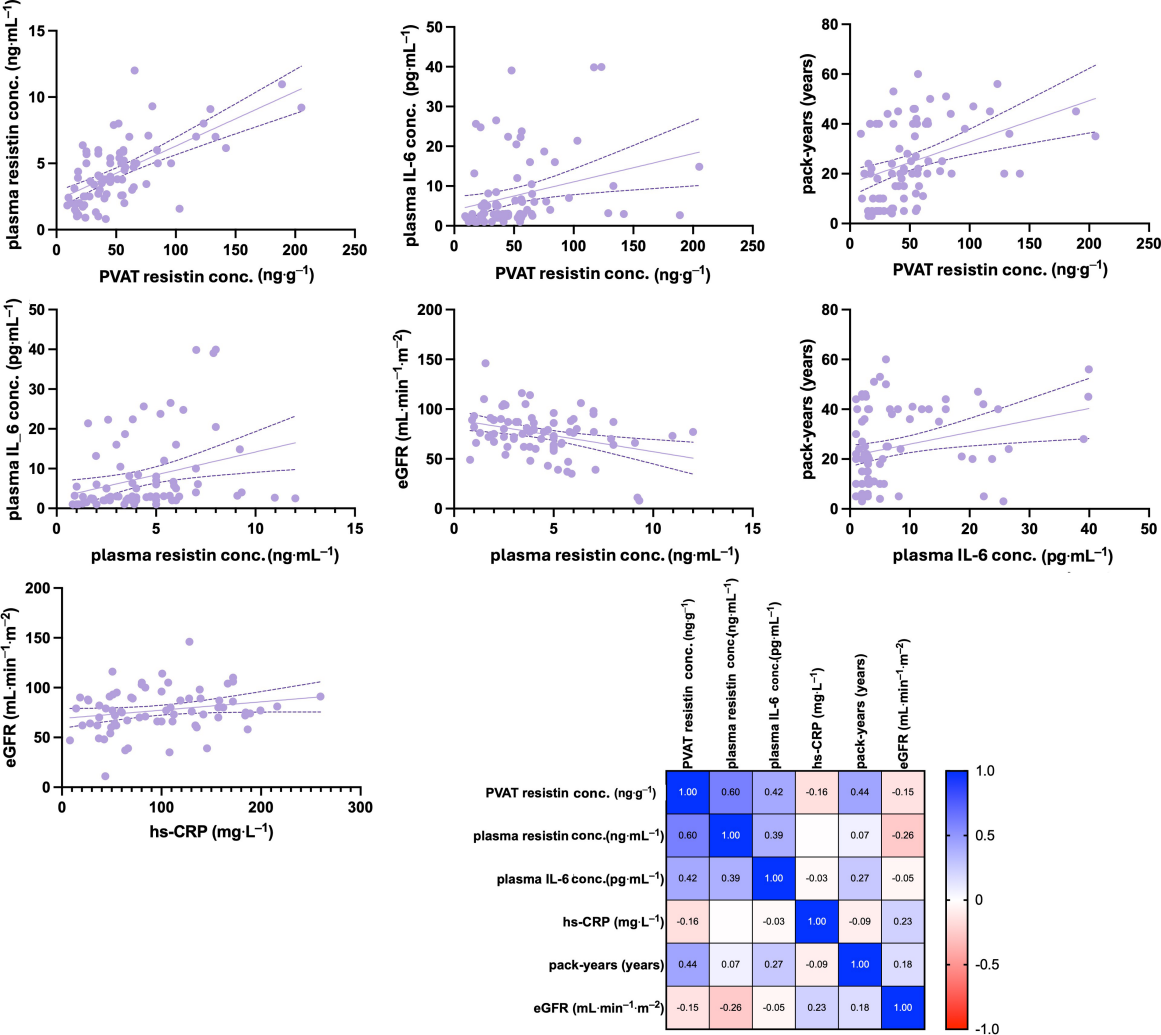
Spearman’s rank correlation analysis of selected clinical parameters in ever-smokers. The matrix with obtained Spearman’s rank correlation coefficient is shown. The concentration of resistin in PVAT showed a strong positive correlation with plasma resistin concentrations and a moderate positive correlation with plasma IL-6 concentrations, as well as with the number of pack-years. Plasma resistin levels exhibited a moderate positive correlation with IL-6 concentrations but a weak negative correlation with eGFR. Additionally, weak positive correlations were observed between plasma IL-6 concentrations and the number of pack-years, as well as between hs-CRP and eGFR concentrations. eGFR, estimated glomerular filtration rate; hs-CRP, high-sensivity C reactive protein; IL-6, interleukin 6; PVAT, perivascular adipose tissue.

There was a strong positive correlation between PVAT and plasma resistin concentrations, (*r* = 0.602, *p* < 0.0001) and between PVAT resistin concentrations and plasma IL-6 concentrations (*r* = 0.4164, *p* < 0.0003). In addition, PVAT resistin concentrations showed a moderate positive correlation with the number of pack-years (*r* = 0.437, *p* < 0.0001). A moderate positive correlation was shown between plasma resistin and IL-6 concentrations (*r* = 0.388, *p* < 0.0008), while a weak negative correlation was demonstrated between plasma resistin concentrations and eGFR (*r* = −0.261, *p* < 0.0001). Weak positive correlations were also noted between plasma IL-6 concentrations and the number of pack-years (*r* = 0.270, *p* < 0.0228) as well as between hs-CRP and eGFR concentrations (*r* = 0.225, *p* < 0.0590). The results are presented in Figure 5.

### 3.3 Multiple regression analysis in ever-smokers

Multiple regression analysis was performed to assess the relationship between PVAT resistin concentrations and other variables. The following variables were shown to be clinically important factors and were included in the final model: the number of pack-years, plasma resistin concentrations, hs-CRP, plasma IL-6 concentrations, eGFR, and the presence of diabetes.

The results indicated that smoking had a significant effect on resistin concentrations in PVAT (adjusted *R*^2^ = 0.28; *β* regression coefficient = 0.5; *p* = 0.0002). Therefore, each additional year of smoking is associated with a 1.12 g/g increase in PVAT resistin concentrations, reflecting a clear exposure-response relationship. Moreover, PVAT resistin concentrations were corelated with plasma resistin concentrations (*R*^2^ = 0.44; *β* regression coefficient = 0.67; *p* < 0.00001). The analysis showed that elevation of the plasma resistin concentrations by 1 mg/L resulted in the rise of PVAT resistin concentrations by 10.7 g/g. Similarly, IL-6 concentrations had a significant effect on PVAT resistin concentrations (*R*^2^ = 0.10; *β* regression coefficient = 0.33; *p* < 0.0004). A 1-unit increase in IL-6 concentrations increased PVAT resistin concentrations by 1.41-unit.

The analysis revealed that plasma resistin concentrations were correlated with the number of pack-years (*R*^2^ = 0.094; *β* regression coefficient = 0.3213; *p* < 0.0007). For each additional pack-year, plasma resistin concentrations increased by 0.053 mg/L. Furthermore, plasma resistin was significantly associated with IL-6 concentrations (*R*^2^ = 0.116; *β* regression coefficient = 0.3521; *p* < 0.0002), with a 0.1-unit change in IL-6 corresponding to a change in plasma resistin concentrations.

## 4 Discussion

The main finding of our study is the presence of higher resistin concentrations in PVAT among cigarette smokers with advanced CAD requiring CABG. These concentrations remain elevated even after a minimum of 1 year after smoking cessation when compared with nonsmokers. Thus, our study demonstrated a significant effect of smoking on PVAT resistin concentrations, resulting in a pronounced proinflammatory state in smokers.

In a previous experimental study, the effect of smoking on adipose tissue was evidenced by a higher expression of genes responsible for producing proinflammatory factors and cytokines such as tumor necrosis factor *α* and IL-6 (23, 24). To our knowledge, our study is the first to show the effect of smoking on resistin concentrations in PVAT. It is noteworthy that in patients with advanced CAD, smoking status was an independent predictor of higher resistin concentrations in PVAT.

Interestingly, the presence of diabetes did not correlate with plasma resistin concentrations in our study. Furthermore, the prevalence of diabetes was similar among never-smokers and past smokers, but lower in current smokers compared with never-smokers and past smokers. This finding is consistent with the study by Wang et al. that showed a negative association between smoking and diabetes (25). However, a large meta-analysis, including 88 prospective cohort studies, indicated that both active and passive smoking were associated with an increased risk of incidences of diabetes (26). It has been established that smoking cessation increases the risk of new-onset diabetes probably due to weight gain (27, 28). At the same time, the fear of weight gain discourages individuals from quitting smoking or encourages them to return to smoking. In the mentioned meta-analysis, smoking cessation significantly reduces the risk of diabetes in the long term but increases it in the short term (26). In our study, BMI was similar in the studied subgroups, and therefore associating weight with a higher prevalence of diabetes in past smokers compared to current smokers was not merited. This may be a result of long-term use of antidiabetic therapy, including the use of metformin or adherence to a diabetic diet. In addition, the awareness of diabetes and the presence of CAD requiring CABG often encourages patients to follow a proper diet and reduce weight while awaiting cardiac surgery.

Since there were no differences in age and gender distribution between our cohorts, the influence of these variables on the tested pro-inflammatory parameters was not revealed. The same applies to hypertension, because all patients in our study had arterial hypertension. In addition, statin administration in all patients allowed a retrospective diagnosis of hypercholesterolemia. However, statin use might explain the lack of differences in TC, HDL-C, and LDL-C concentrations between ever-smokers and never-smokers. Our patients underwent elective CABG, which is typically performed a few weeks after referral. Statin treatment may have been started at the time of referral, or statin dose may have been increased, especially among smokers. However, previous research demonstrated that smoking reduces HDL-C concentrations, while its effect on LDL-C concentrations is corelated with various metabolic disorders, including obesity (29–31). The effect of lipolysis in smokers could lead to a decrease in LDL-C concentrations, which is not necessarily beneficial (32). This is consistent with our finding that TC and LDL-C concentrations are lower in current smokers.

Some studies demonstrated a trend among smokers toward elevated eGFR and a higher prevalence of proteinuria, which contributed to the progression of renal function decline (33, 34). It is worth noting that despite the trend toward higher eGFR in smokers in our study, a negative correlation between serum resistin and eGFR was identified. This finding may suggest a pathomechanism underlying the detrimental effects of smoking on renal function.

Another important finding is the linear relationship between resistin concentrations in plasma and PVAT, indicating a mutual influence between these compartments. However, the cause-and-effect relationship could not be determined. It is possible that PVAT is highly sensitive to the effects of smoking, and the smoking-induced proinflammatory state in PVAT may spread to the plasma through various mechanisms. It is noteworthy that there is a higher resistin concentrations in PVAT in current smokers compared to past smokers, but a lower concentrations of resistin in plasma in current smokers than in past smokers. Additionally, a significant dose-dependent effect was observed for resistin concentrations in both PVAT and plasma, with a more pronounced effect noted in PVAT. The influence of smoking exposure on IL-6 and hs-CRP was less pronounced. Furthermore, the correlation between resistin concentrations in PVAT and plasma was stronger than that observed for other pro-inflammatory markers, such as IL-6 or hs-CRP in plasma. This dose-dependent effect of smoking on resistin concentration suggests that resistin may be the most reliable biomarker for smoking exposure and the associated damage.

Previous research showed an association between plasma resistin concentrations and smoking in the general population and in patients with stable CAD, as well as an association with pack-years of smoking (35, 36). This is consistent with our observation in patients with confirmed advanced CAD regarding resistin concentrations in plasma and PVAT. However, its association with pack-years was less pronounced in plasma than PVAT concentrations. This was also observed for plasma inflammatory markers, such as IL-6 and hs-CRP concentrations, which were higher in current smokers.

PVAT inflammation plays a critical role in the progression of atherosclerotic plaque (37). Our study showed that smoking is associated with a higher proinflammatory state that persists for at least 1 year after smoking cessation, despite the presence of other risk factors for atherosclerosis. These findings highlight the significant role of smoking in sustaining a proinflammatory environment conducive to the development of atherosclerosis. Epidemiological evidence consistently links cigarette smoking to higher rates of adverse events and mortality in smokers compared with nonsmokers (38). In addition, smoking in the presence of other atherosclerotic risk factors significantly increases mortality. Data from a prospective study of the Finnish population, with and without diabetes, demonstrated an increased risk of mortality from CAD among smokers (39). In our previous study, we identified an association between elevated resistin concentrations in PVAT and increased mortality among patients after CABG (40). In this context, our findings highlight the potential role of resistin as a diagnostic and therapeutic parameter in the management of atherosclerosis.

### 4.1 Clinical implication and future study perspectives

Resistin shows potential as a biomarker for smoking-induced inflammation, with strong correlations observed between its plasma concentration and PVAT, which interacts with the arterial wall in the development of atherosclerotic plaque. Measuring its plasma concentration may help identify individuals at high risk for cardiovascular events, both during active smoking and after cessation. Additionally, elevated resistin concentration may predict poorer outcomes following CABG, suggesting its utility in risk stratification and targeted therapies. Novel treatments targeting resistin could complement smoking cessation programs.

Future studies should focus on the long-term effects of smoking cessation, with prolonged follow-up to determine if resistin concentration eventually normalize. Investigations into resistin and other adipocytokines may provide further insights into the role of adipose tissue in smoking-related inflammation. Furthermore, therapeutic interventions, including anti-inflammatory treatments such as anti-resistin agents, should be evaluated to mitigate persistent inflammation in former smokers.

### 4.2 Limitations

Our study has several limitations. First, it did not include a control group of patients without coronary artery atherosclerosis who underwent cardiac surgery. Inclusion of such a group could help establish normal serum and PVAT resistin concentrations and correlations with other parameters in the absence of CAD. Second, due to the small sample size, our study was limited to 1 year after smoking cessation. A larger study group would allow a longer follow-up of resistin concentrations after smoking cessation. Finally, the measurement of the concentrations of other adipocytokines in PVAT would allow evaluation of the effect of smoking on adipose tissue inflammation.

## 5 Conclusion

The proinflammatory state observed in patients with confirmed advanced CAD, as assessed based on resistin concentrations in plasma and PVAT, is strongly associated with smoking. The proinflammatory state often persists even 1 year after smoking cessation, as evidenced by serum and PVAT resistin concentrations and their association with the number of pack-years of smoking.

## Data Availability

The raw data supporting the conclusions of this article will be made available by the authors, without undue reservation.
